# Exosomes secreted by ATF3/Nrf2-mediated ferroptotic renal tubular epithelial cells promote M1/M2 ratio imbalance inducing renal interstitial fibrosis following ischemia and reperfusion injury

**DOI:** 10.3389/fimmu.2025.1510500

**Published:** 2025-02-05

**Authors:** Qiao Tang, Jiatao Xie, Yifei Wang, Chong Dong, Qian Sun

**Affiliations:** ^1^ Department of Anesthesiology, Renmin Hospital of Wuhan University, Wuhan, China; ^2^ The First Clinical College of Wuhan University, Wuhan, China; ^3^ Organ Transplantation Center, Tianjin First Central Hospital, Tianjin, China; ^4^ Tianjin Key Laboratory for Organ Transplantation, Tianjin, China

**Keywords:** ischemia and reperfusion, renal interstitial fibrosis, ferroptosis, activation transcription factor 3, exosome, macrophages

## Abstract

**Background:**

Severe renal ischemia and reperfusion injury (IRI) progresses to renal interstitial fibrosis (RIF) with limited therapeutic strategies. Although ferrptosis and macrophage polarization both play important roles in this model, their specific pathogenesis and interactions have not been elucidated. Therefore, we aimed to explore the mechanisms by which ferrotosis occurs in renal tubular epithelial cells (RTECs) and ferroptotic cell-derived exosomes induce macrophage polarization in IRI-related RIF model.

**Methods:**

*In vivo*, C57BL/6J mice were randomly divided into four groups: sham group, ischemia and reperfusion (IR) group, IR + Ferrostatin-1 (Fer-1) group, and IR +ATF3 knockdown (ATF^KD^) group. *In vitro*, RTECs were divided into control (CON) group, hypoxia/reoxygenation (HR) group, HR +Fer-1 group, HR + siRNA-ATF3 (siATF3) group.

**Result:**

Compared with the sham group, the IR group showed more severe kidney injury in HE staining, more collagen fibers in Masson staining, and higher α-SMA expression levels in immunohistochemistry. Total iron and MDA content increased while GSH content decreased. The IR group had more significant mitochondrial damage and higher PTGS2 and TFRC mRNA levels than those in the sham group. Compared with the IR group, the above indexes were all alleviated in the IR+Fer-1 or IR+ATF3^KD^ groups. In addition, the protein expressions of ATF3, Nrf2 and HO-1 in the IR group were increased than those in sham group. Compared with the IR group, ATF3 expressions in the IR+Fer-1 or IR+ATF3^KD^ groups were decreased, and the protein contents of Nrf2 and HO-1 were further increased. Moreover, there were higher levels of M2 markers (Arg1, TGF-β and IL-10 mRNA) in the IR group than those in the sham group, and lower levels in the IR+Fer-1 group or in the IR+ATF3^KD^ group compared with the IR group. The results of *in vitro* experiment are consistent with those of *in vivo* experiment. Mechanistically, the release of exosomes carrying miR-1306-5p by the HR group promoted more M2 macrophage.

**Conclusion:**

ATF3 might accelerate the ferroptosis by inhibiting Nrf2/ARE pathway, and exosomes from ferroptotic cells reduced the M1/M2 macrophage ratio, promoting fibrosis.

## 1 Introduction

Severe renal ischemia and reperfusion injury (IRI) is a risk factor for the development and progression of chronic kidney disease (CKD) and end-stage renal disease, which will further increase the global morbidity and mortality (1). Long-term IRI causes persistent non-specific inflammation, drives immune cell infiltration and cytokine release, and causes excessive accumulation of extracellular matrix, which leads to irreversible renal interstitial fibrosis (RIF) and decreases the long-term survival rate of transplanted kidney (2, 3). Persistent inflammation and RIF are two pathophysiological processes involved in the maladaptive repair process of IRI to CKD transformation (4, 5). During IRI-related RIF, severe and persistent inflammatory injury leads to excessive repair of M2 macrophages, which not only releases more pro-fibrotic factors to further activate myofibroblasts, but also encourages macrophages to directly transform into myofibroblasts (6). Therefore, macrophage polarization and its functional diversity are important factors of IRI-related RIF in transplanted kidney.

Activation transcription factor 3 (ATF3) is a basic leucine zipper (bZIP) DNA binding protein induced by oxidative stress and a member of the transcription factor family of ATF/cAMP responsive element binding proteins. In recent years, studies on fibrosis of heart, liver, lung and other organs have shown that ATF3 could accelerate extracellular matrix accumulation and down-regulation of ATF3 expression could ameliorate fibrosis (7–9). Although Takumi Yoshida et al. demonstrated that renal IRI and oxidative stress during this period significantly enhanced ATF3 expression in the acute phase (10), there is a lack of research in the field of severe IRI on interstitial fibrosis of transplanted kidneys in the chronic phase. ATF3 was initially identified through our previous analysis of ferroptosis-related genes before and after renal ischemia and reperfusion (11), which confirmed that ATF3 is highly expressed following renal IRI. Building on this, we further discovered that ATF3 is significantly upregulated in renal IRI-related renal interstitial fibrosis. This finding led us to initiate a focused investigation into the role of ATF3 in this pathological process. Notably, ATF3 upregulation restricts the activation of Nrf2 signaling pathway, thus aggravating ferroptosis (12), which may be due to the fact that ATF3-Nrf2 heterodimer formed on the promoter of Nrf2 target gene ARE directly inhibits the antioxidant stress ability of Nrf2 (13). In addition, renal tubular epithelial cells (RTECs) are considered to be the most sensitive cells to ferroptosis in the kidney, and are usually the most serious parenchymal cells of IRI (14, 15). Inhibition of ferroptosis in RTECs is helpful to alleviate RIF (16), so it’s suspected that ATF3 may promote ferroptosis in RTECs by inhibiting Nrf2 signaling pathway in IRI-related RIF of transplanted kidney.

Exosomes is an important regulator of transplanted kidney and immune system, and promote the repair and fibrosis of kidney tissue suffering from IRI (17). Intriguingly, Dai EY et al. proved for the first time in 2020 that there is a connection between ferroptotic cells and macrophages, that is, the exosomes released by ferroptotic cancer cells carry KRAS, which led to M2 macrophages polarization (18). Subsequently, in 2022, Qiying Chen et al. further proved that exosomes derived from ferroptotic cardiomyocytes affected M1 macrophages polarization, contributing to the pathological progression of myocardial infarction (19). Although the exosomes secreted by RTECs can promote RIF following IRI (20), it is still unknown whether exosomes derived from ferroptotic RTECs can induce macrophage polarization and the internal mechanism. Hence, this study aimed to investigate that ATF3 promoted ferroptosis in RTECs by inhibiting Nrf2 pathway, and the exosomes derived from ferroptotic RTECs induced M1/M2 ratio imbalance, offering a potential therapeutic target for decelerating the progression of renal IRI towards RIF.

## 2 Methods

### 2.1 RIF mouse model associated with IRI

Six to eight weeks C57BL/6 mice (Experimental Animal Center, Renmin Hospital of Wuhan University, No. 430727230101780713) were housed alone in an animal laboratory without specific pathogens, with a temperature of 22-25℃, a relative humidity of 60-65% and a light/dark cycle of 12 hours (free to eat and drink). The experimental protocol of the present study was approved by the Ethics Committee of Renmin Hospital of Wuhan University and in accordance with the principles of Laboratory Animal Care by the National Institutes of Health (permit no. 20210124). Mice were adapted for a week before the experiment, and then randomly divided into four groups (n=5): sham group, ischemia and reperfusion (IR) group, IR+Fer-1 group and IR+ATF3 knockdown (ATF3^KD^) group. 1% pentobarbital sodium was injected intraperitoneally to anesthetize mice. After the midline of the abdomen was cut to expose the abdomen, two renal pedicles were separated and clamped with capillary clamps. After 30 minutes, the capillary clamps were removed from the renal pedicles for reperfusion. During ischemia, the body temperature was maintained at 37-38°C by using a heating blanket with steady-state control through a rectal temperature probe. After sewing the incision, the mice were allowed to eat at will. The mice in the sham group received the same operation but did not use capillary forceps. 100 μL adenovirus carrying sh-ATF3 was injected through the tail vein with a titer of 1 × 10^11^ TU. Fer-1 was injected intraperitoneally at a dose of 5 mg/kg/day. On the 14th day of IR, mice were euthanized under the anesthesia of 1% pentobarbital sodium. Some renal tissues were fixed in 4% paraformaldehyde, and the rest were frozen at -80°C.

### 2.2 Measurement of blood urea nitrogen and serum creatinine levels

1 ml blood was centrifuged at 3,000 × g for 10 mins at 4°C, and then serum was separated and stored at -20°C. serum creatinine and BUN were measured using an Olympus automatic analyzer (Chemray 800; Rayto Life and Analytical Sciences).

### 2.3 Hematoxylin-eosin staining

Tissue was fixed in buffered 4% formalin solution for 24 hours and embedded in paraffin. Then, 4μm thick slices were dewaxed in xylene, rehydrated with gradient ethanol, and stained with hematoxylin for 5 minutes. Next, 1% hydrochloric acid-ethanol was used to distinguish the slices for 3 s, and 5% eosin was used to dye for about 3 minutes, then dehydrated, removed and embedded. Finally, the slices were observed under a microscope. HE staining was evaluated for renal tubular injury based on brush border loss, vacuolization, cellular desquamation, tubular dilatation, and tubular degeneration (all scored on a scale ranging from 0-3), with a maximum tubular injury score of 15 (9).

### 2.4 Immunohistochemical staining

Paraffin-embedded renal tissue slices with a thickness of 4μm were heated in EDTA buffer for 20 minutes for antigen recovery. Next, the slices were treated with 0.3% H_2_O_2_ for 10 minutes to eliminate endogenous peroxidase activity, and then washed with PBS. After blocking with 5% BSA for 20 minutes, the slices were incubated with the primary antibody against α-SMA (1: 1000, GB12044, Servicebio) at 4°C overnight. After that, the sections were incubated with HRP Goat Anti-Mouse lgG (1:3000, GB23301, Servicebio) for 1 hour. Finally, the slices were developed with 3,3’- diaminobenzidine tetrahydrochloride for 3 minutes and analyzed under a microscope. Quantitative analysis of immunohistochemistry was completed by two researchers who were unaware of the experimental conditions using ImageJ software to analyze five high magnification fields of view of each randomly selected mouse (21).

### 2.5 Masson staining

Paraffin-embedded renal tissue slices with a thickness of 4μm were dewaxed and hydrated, and then stained with iron hematoxylin after overnight with potassium dichromate. Next, ponceau acid fuchsin-phosphomolybdic acid dyeing and aniline blue dyeing were carried out, and finally the film was dehydrated and sealed. Microscopic examination showed that the collagen fibers were dyed blue and the nucleus was dyed blue-brown. The positive blue tissue in each image was quantitatively analyzed by ImageJ software (22).

### 2.6 Mouse renal tubular epithelial cell line cells culture and treatment

RTECs were carried out according to the following protocol: Control group (CON), hypoxia/reoxygenation (HR) group, HR +Fer-1 group, HR + siATF3 group. RTECs was cultured in 5% CO_2_ incubator at 37°C, supplemented with 10% fetal bovine serum in Dulbecco modified Eagle medium. In HR experiment, RTECs were exposed to serum-free medium for 48 hours of hypoxia (5%CO_2_, 1%O_2_ and 94%N_2_), and replaced with normal medium for 48 hours of reoxygenation (5%CO_2_, 21%O_2_ and 74%N_2_). It was then incubated with Fer-1(10μM) for 24 hours. For ATF3 silencing, RTECs grown under normal conditions were transfected with siATF3 according to the manufacturer’s guidelines.

### 2.7 Cell activity assay

RTECs with 5×10^3^ cells/well were inoculated on a 96-well plate, CCK8 reagent was added according to the manufacturer’s requirements, and incubated at 37°C for 4 hours.

### 2.8 Exosomes extraction

Extracellular vesicles were extracted by ultracentrifugation. Cultivate RTECs using serum without extracellular vesicles, collect the supernatant, centrifuge at 10, 000 g for 30 minutes to remove fragments, then centrifuge at 100, 000 g for 120 minutes in an ultracentrifuge. Discard the supernatant, collect extracellular vesicles from the sediment, and resuspend in PBS. Store in a refrigerator at -80°C. All steps were carried out in a 4°C; environment.

### 2.9 Nanoparticle tracking analysis

Resuspension the exosomes in PBS at a concentration of 5 mg/L, and further dilute the suspension by 100-500 times. Manually inject the sample into the sample chamber at ambient temperature. Each sample is equipped with a 488 nm laser and a high sensitivity sCMOS camera. By collecting scattered light signals from nanoparticles, an image of the Brownian motion of the nanoparticles in the solution is captured to track the Brownian motion of each particle. Finally, calculate the radius and concentration of exosomes using NTA analysis software.

### 2.10 Exosome uptake assay

The green fluorescent dye PKH67 (UR52303, Umibio) was co-cultured with exosomes from RTECs cells to label exosomes. Macrophages were adhered to laser confocal dishes and co-cultured with PKH67-labeled exosomes for 2 hours. RAW 264.7 cells were incubated dropwise with DAPI for 5 min away from light for nuclear staining, and RAW 264.7 cellular uptake of exogenous exosomes was detected by laser confocal microscopy (Olympus, UltraVIEW VoX & IX81).

### 2.11 miRNA-seq analysis in exosomes derived from RTECs

Differentially expressed miRNAs in exosomes were screened via the edgeR package (version 4.0.16) in the R software (version 4.3.1) with a critical criterion of |log2FoldChange| > 1 and *P* < 0.05, and were demonstrated by volcano plotting with the ggplot2 package (version 3.5.0). Their expression levels were demonstrated by heatmaps drawn with the pheatmap package (version 1.0.12). Intersections were taken for target genes predicted by miranda, RNAhybrid and miRWalk and visualized by the VennDiagram package (version 1.7.3). Finally, gene ontology (GO) annotation analysis and Kyoto Encyclopedia of Genes and Genomes (KEGG) pathway enrichment analysis were performed on the intersected target genes via the clusterProfiler package (version 4.10.1).

### 2.12 Flow cytometry

After exosome incubation, RAW 264.7 cells were centrifuged at 350g for 5 min to remove medium and resuspended in PBS. RAW 264.7 cells were then incubated with CD86 antibody (ab239075, abcam) or with CD206 antibody (18704-1-AP, proteintech) for 30 min at 4°C away from light. Then incubated with Alexa Fluor 488 - conjugated Affinipure Goat Anti-Rabbit IgG (H+L) for 30min at 4°C away from light. After centrifugation and resuspension, RAW 264.7 cells were analyzed by CytoFLEX LX flow cytometer (Beckman, USA).

### 2.13 Detection of total iron content, malondialdehyde and glutathione

Tissue iron assay kit (A039-2-1, Nanjing Jiancheng Bioengineering Institute, China) was applied for iron content assay. According to this scheme, kidney tissue was homogenized in phosphate buffered saline, and then iron chromogenic agent was added. After vortexing, the mixture was clarified by centrifugation at 1500 g for 10 minutes. Finally, the absorbance was measured at the wavelength of 520 nm. Similar to iron content assay, MDA (A003-1-1) and GSH (A006-2-1) assay kit were both built in Nanjing Jiancheng Bioengineering Institute and carried out under the manufacturer’s instructions. The results were normalized by protein content.

### 2.14 Transmission electron microscope

Kidney tissues from mice were harvested and fixed with 5.4% glutaraldehyde at 2°C overnight, and then fixed with 1% osmium tetroxide at 2°C for 37 hours. Acetone gradient (50%, 70%, 90% and 100% respectively) was dehydrated and then embedded in epoxy resin. The ultrathin sections were dyed with uranyl acetate/lead citrate for visualization by TEM.

### 2.15 Western blot

Total protein was obtained from renal tissue by lysis buffer and its concentration was measured by Bicinchoninic Acid Protein Kit. Protein samples were separated on sodium dodecyl sulfate-polyacrylamide gel and transferred to polyvinylidene fluoride membrane, then blocked with 5% BSA and incubated with β-actin antibody (1: 1000, Servicebio, GB11001-100, China), ATF3 antibody (1: 1000, CST, 33593S, USA), Nrf2 antibody (1: 1000, protein, 16396-1-AP, USA). After washing with TBST for 30 minutes, PVDF membrane was incubated with HRP conjugated Goat Anti-Rabbit IgG (1: 3000, Servicebio, GB23303, China) for 1 hour at room temperature. ECL reagents were applied to generate chemiluminescence signals, which were detected by ChemiDoc™ system (Bio-Rad, USA). The image is used to analyze the intensity of protein bands.

### 2.16 Quantitative real-time PCR

Total RNA was extracted from renal tissue and complementary DNA was synthesized by reverse transcription kit. Finally, RT-qPCR was carried out on Lightcycler 480II real-time fluorescence quantitative PCR system (Roche, Germany). The β-actin is used for expression standardization. The primers are listed in **Table 1**.

**Table 1:** Gene-specific primers used for RT-qPCR.

Gene	Forward primer	Reverse primer
TFRC	CTTCCTGTCGCCCTATGTATCT	TCCCTGAATAGTCCAAGTAGCC
PTGS2	GTGATGAGCAACTATTCCA	GGTGAATGACTCAACAAAC
iNOS	TTGGCTCCAGCATGTACCCT	TCCTGCCCACTGAGTTCGTC
IL-1β	TCAGGCAGGCAGTATCACTC	AGCTCATATGGGTCCGACAG
TNF-α	CGTCAGCCGATTTGCTATCT	CGGACTCCGCAAAGTCTAAG
Arg1	CAGAGTATGACGTGAGAGACCA	GCATCCACCCAAATGACACATA
IL-10	GCTGGACAACATACTGCTAACCG	CACAGGGGAGAAATCGATGACAG
COL1α1	AAGAAGCACGTCTGGTTTGGAG	GGTCCATGTAGGCTACGCTGTT
FN1	AAGGCTGGATGATGGTGGACT	TCGGTTGTCCTTCTTGCTCC
β-Actin	GTGGGAATGGGTCAGAAGGA	TCATCTTTTCACGGTTGGCC

The relative expression of genes was detected by 2^- ΔΔCT^ method.

### 2.17 Statistical analysis

Data are presented as the means ± standard deviation (SD). GraphPad Prism 9.0 were for statistical analysis and *P* < 0.05 was considered statistically significant. Student’s t-test was used to compare with two groups and ANOVA followed by Tukey’s test was for multiple-group comparisons. The Kruskal-Wallis test was applied to analyze data that does not follow a normal distribution. All experiments and assays were independently repeated at least three times.

## 3 Results

### 3.1 RIF evolved from severe bilateral IRI

**Figure 1:**
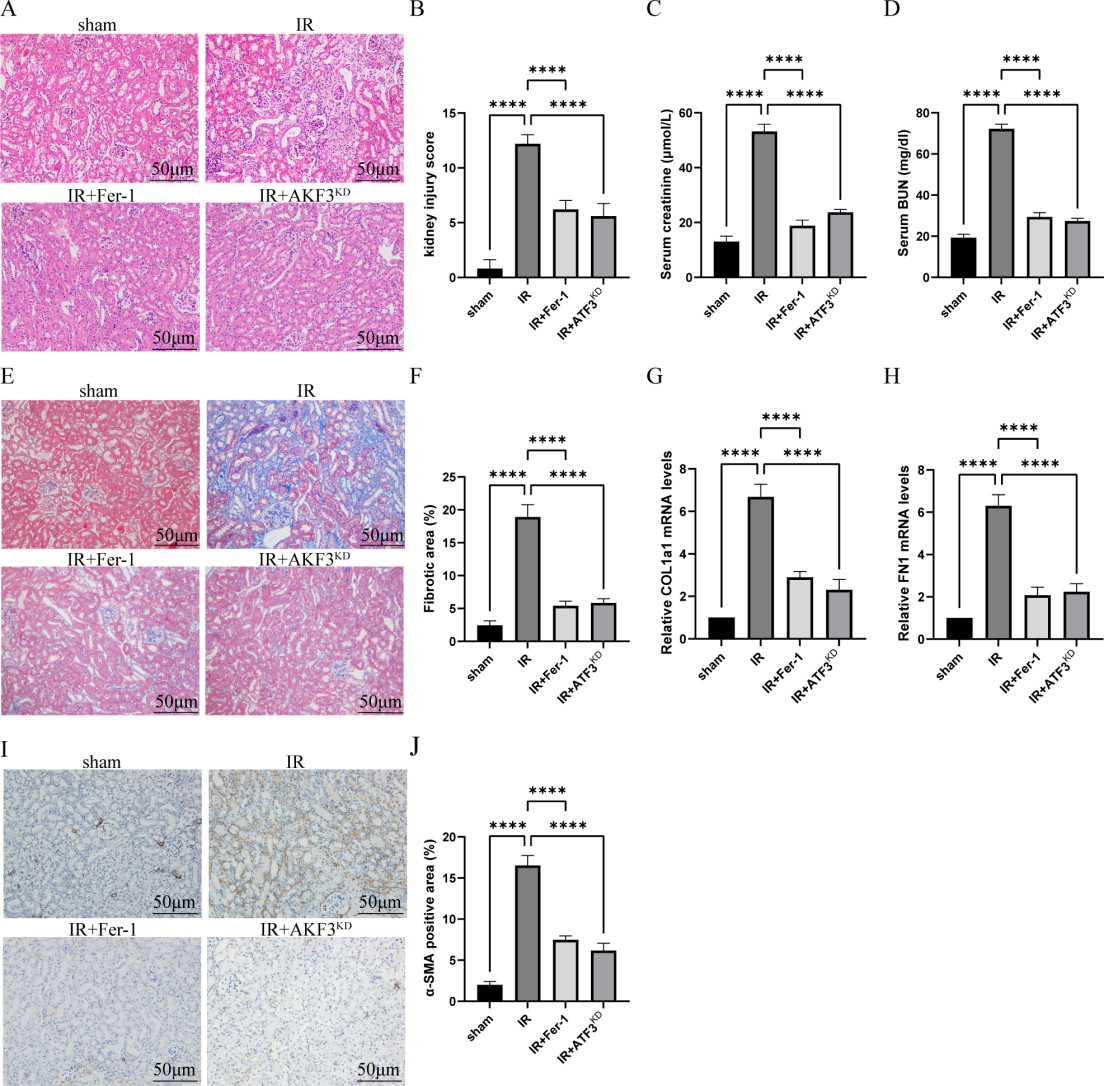
Establishment of RIF model following severe bilateral IRI. **(A)** Representative HE staining images of kidneys for the assessment of IRI. The scale bar represents 50 μm. **(B)** Pathologic scoring of renal HE staining. **(C, D)** Serum creatinine and BUN levels. **(E)** Representative Masson staining images of kidneys to evaluate the area size of collagen fibers. The scale bar represents 50 μm. **(F)** Quantification of Masson staining. **(G, H)** Relative level of mRNA expression of COL1a1 and FN1 in the kidneys detected by RT-qPCR. The mRNA level of sham group was normalized as 1. **(I)** Representative Immunohistochemistry staining images of α-SMA expression level in the kidneys. The scale bar represents 50 μm. **(J)** Quantification of α-SMA positive cells in IHC. Data are expressed as means ± SD, n=5, ^****^P < 0.0001.

As shown in **Figures 1A, B**, compared with sham group, HE staining in IR group indicated renal tubular dilatation, renal tubular epithelial injury and lumen destruction, accompanied by immune cell infiltration, while renal injury in IR+Fer-1 and IR+ATF3^KD^ groups was alleviated. Meanwhile, compared with sham group, serum creatinine and BUN were significantly higher in the IR group, while both were significantly lower in the IR+Fer-1 and IR+ATF3^KD^ groups (**Figures 1C, D**). These results imply that renal tissue damage still exists in the 14days following IR and can be ameliorated by ferroptosis inhibitors.

As shown in **Figures 1E, F**, histological analysis of masson staining revealed that there were obvious extracellular matrix deposition and fibrosis formation in IR group. The mRNA levels of COL1a1 and FN1 in the IR group were increased than those in the sham group (**Figures 1G, H**). As shown by immunohistochemistry in **Figures 1I, J**, α-SMA, a myofibroblast marker, was expressed more in IR group than in sham group. Compared with IR group, the above fibrosis indexes were significantly reduced in IR+Fer-1 and IR+ATF3^KD^ groups. In conclusion, the successful establishment of a RIF model with 30 minutes of ischemia and 14 days of reperfusion is available for further study, which was ameliorated by ferroptosis inhibitors.

### 3.2 Ferroptosis occurred in IRI-related RIF model

**Figure 2:**
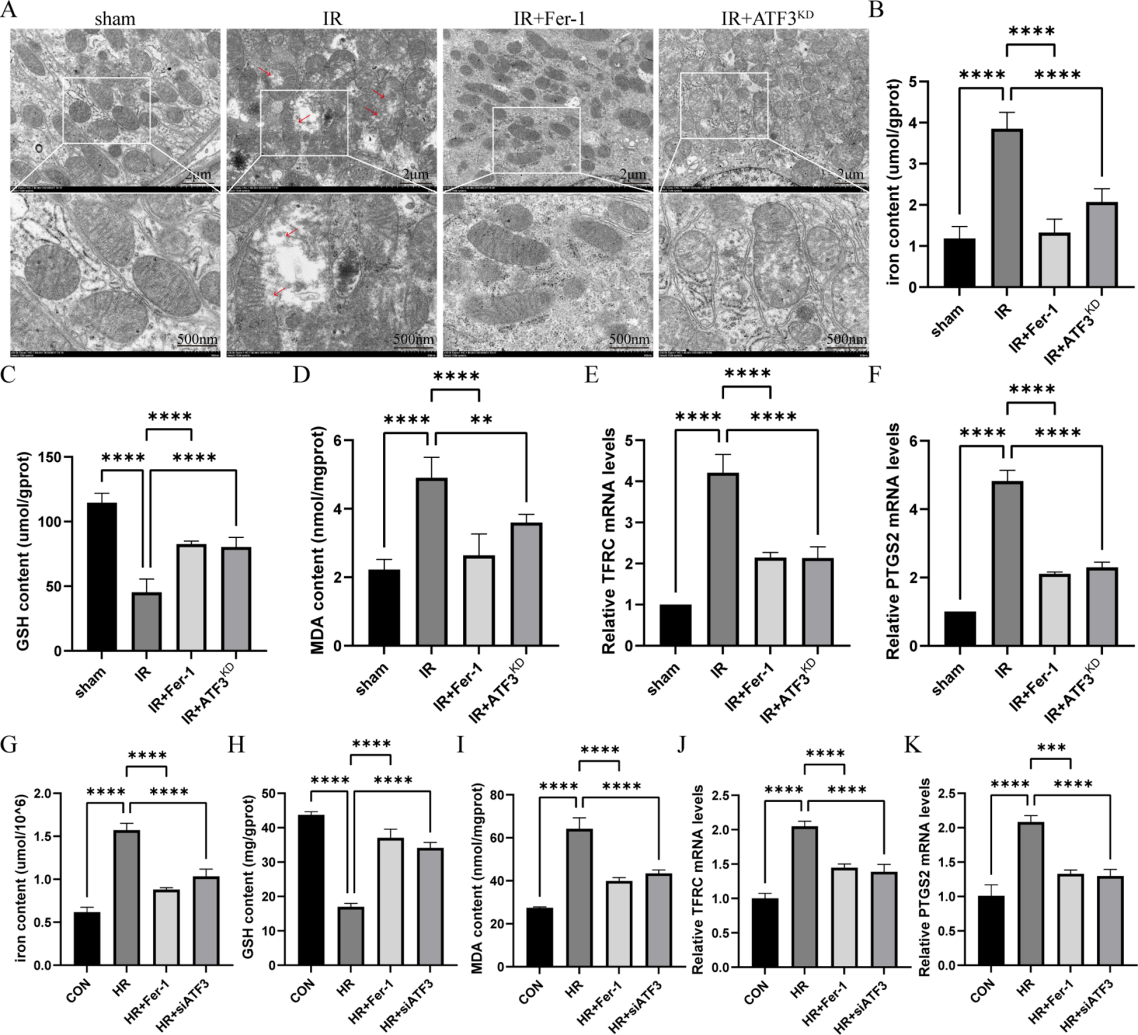
Ferroptosis in renal IRI-related RIF model. **(A)** Representative pictures acquired by transmission electron microscopy. The scale bar represents 2 μm and 500nm. **(B-D)** Total iron, GSH and MDA content in renal tissue. **(E, F)** Relative level of mRNA expression of TFRC and PTGS2 in the kidneys detected by RT-qPCR. The mRNA level of sham group was normalized as 1. **(G-I)** Total iron, GSH and MDA content in RTECs. **(J, K)** Relative level of mRNA expression of TFRC and PTGS2 in RTECs detected by RT-qPCR. Data are expressed as means ± SD, n=5, ^**^P < 0.01, ^****^P < 0.0001.

Compared with sham group, mitochondrial crista disappeared, and outer membrane ruptured in IR group, but it was obviously ameliorated in IR+Fer-1 and IR+ATF3^KD^ groups (**Figure 2A**). Next, iron content was significantly increased in the IR group compared to the sham group, and Fer-1 treatment significantly reduced abnormal iron accumulation, as the same as IR+ATF3^KD^ groups (**Figure 2B**). A decrease of GSH levels and an increase of MDA content were observed in the IR group compared to the sham group (**Figures 2C, D**). A decrease in MDA content and an increase in GSH levels were observed in IR+Fer-1 and IR+ATF3^KD^ groups compared with the IR group. In addition, the mRNA expression levels of PTGS2 and TFRC, the molecular markers of ferroptosis, were significantly upregulated in IR group but were limited in IR+Fer-1 and IR+ATF3^KD^ groups, suggesting that ferroptosis occurred in IRI-related RIF model (**Figures 2E, F**). *In vitro* experiments showed the same trend for the above mortality parameters (**Figures 2G–K**). In summary, the above results demonstrate that ferroptosis takes place among renal RTECs in IRI-related RIF model.

### 3.3 ATF3/Nrf2 pathway regulates ferroptosis *in vivo and in vitro*

**Figure 3:**
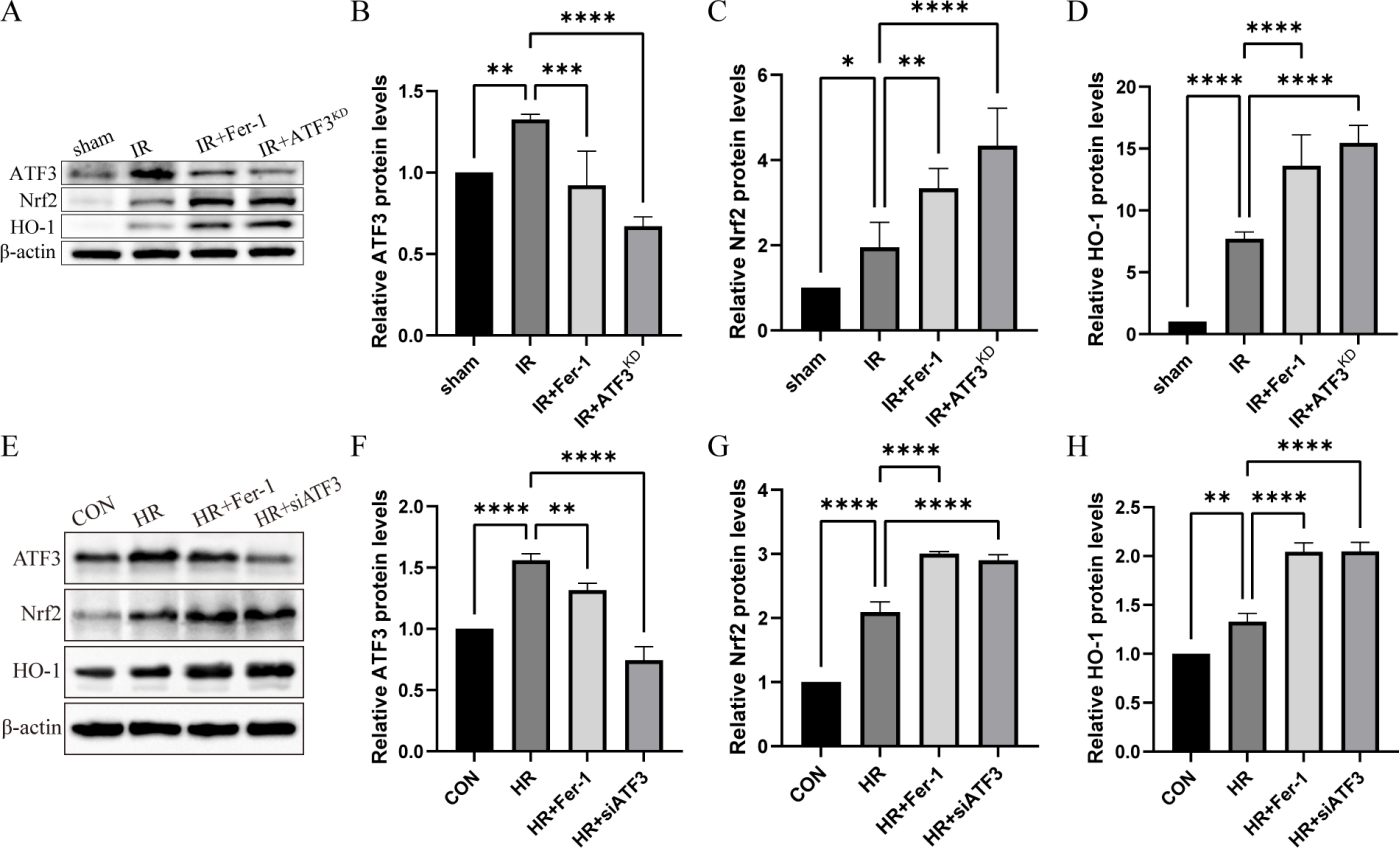
ATF3/Nrf2 pathway regulates ferroptosis in RTECs in the renal IRI-related RIF model. **(A)** Relative level of protein expression of ATF3, Nrf2 and HO-1 in the kidneys detected by Western blot. **(B-D)** Quantification of ATF3, Nrf2 and HO-1 protein expression in the kidneys detected by Western blot. **(E)** Relative level of protein expression of ATF3, Nrf2 and HO-1 in RTECs detected by Western blot. **(F-H)** Quantification of ATF3, Nrf2 and HO-1 protein expression in RTECs detected by Western blot. The protein level of CON group was normalized as 1. Data are expressed as means ± SD, ^*^P < 0.05, ^**^P < 0.01, ^***^P < 0.001, ^****^P < 0.0001.

*In vivo*, the increase of ATF3 expression was identified in the RIF mouse model (**Figures 3A–D**). Similarly, the expression levels of Nrf2 and its downstream gene HO-1 in IR group were increased compared with those in sham group. In IR+Fer-1 and IR+ATF3^KD^ groups, the expression of ATF3 decreased, while the expression of Nrf2 and HO-1 further increased. *In vitro* experiments also revealed that the expression levels of ATF3, Nrf2 and HO-1 in HR group were higher than those in CON group, but the expression levels of ATF3 in HR+Fer-1 or HR+siATF3 group were lower than those in HR group, and the expression levels of Nrf2 and HO-1 were further increased (**Figures 3E–H**). These results indicate that ferroptosis in RTECs during RIF following renal IRI was mediated by ATF3 inhibiting the Nrf2/ARE pathway.

### 3.4 Ferroptosis promotes M2 macrophage polarization in IRI-related RIF model

**Figure 4:**
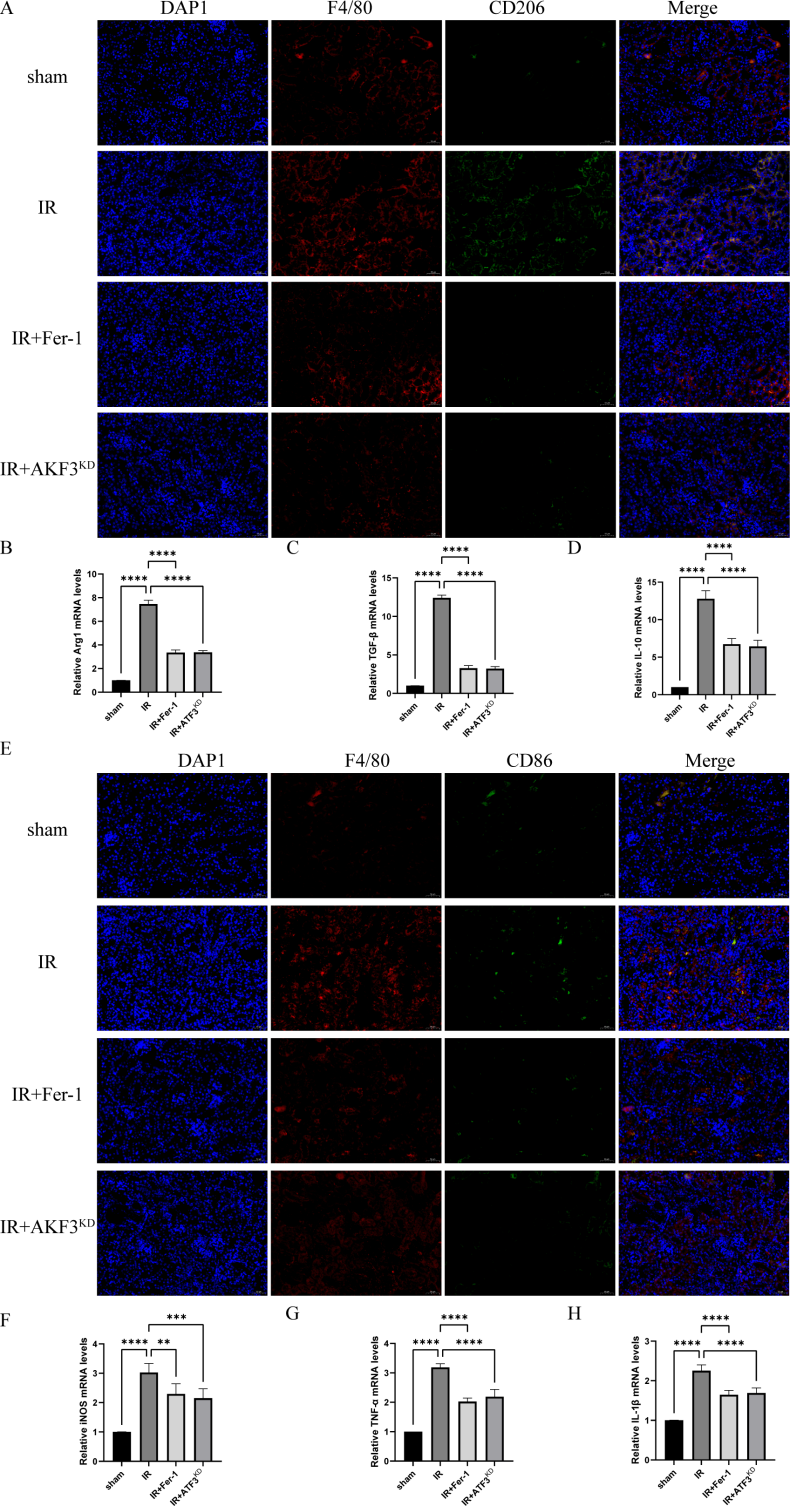
Ferroptosis promotes polarization of M2 macrophages in renal IRI-related RIF model. **(A)** Representative images of immunofluorescence co-staining of F4/80 (red signal) and CD206 (green signal) in the kidneys. Cell nuclei were stained with DAPI (blue signal). The scale bar represents 50 μm. **(B-D)** Relative level of mRNA expression of Arg1, TGF-β, and IL-10 in the kidneys detected by RT-qPCR. **(E)** Representative images of immunofluorescence co-staining of F4/80 (red signal) and CD86 (green signal) in the kidneys. Cell nuclei were stained with DAPI (blue signal). The scale bar represents 50 μm. **(F-H)** Relative level of mRNA expression of iNOS, TNF-α, and IL-1β in the kidneys detected by RT-qPCR. The mRNA level of sham group was normalized as 1. Data are expressed as means ± SD, ^**^P < 0.01, ^***^P < 0.001, ^****^P < 0.0001.

Immunofluorescence of CD206 (surface marker of M2 macrophages) showed a significant accumulation of M2 macrophages in the IR group compared to the sham group and a significant reduction of M2 macrophages in the IR+Fer-1 and IR+ATF3^KD^ groups compared to the IR group (**Figure 4A**). Although the trend of CD86 (surface marker of M1 macrophages) was similar to that of CD206, the magnitude of change was not as large as that of M2 macrophages (**Figure 4E**). As shown in **Figures 4B–D**, RT-qPCR analysis revealed that M2 markers (Arg1, TGF-β, and IL-10) showed the same trend as CD206. typical M1 markers (iNOS, TNF-α, and IL-1β) showed the same trend as CD86 (**Figures 4F, H**). Taken together, these data suggested that ferrotosis might accelerate the transformation of macrophages into M2 in renal IRI-related RIF.

### 3.5 Characterization of exosomes derived from ferroptotic RTECs

**Figure 5:**
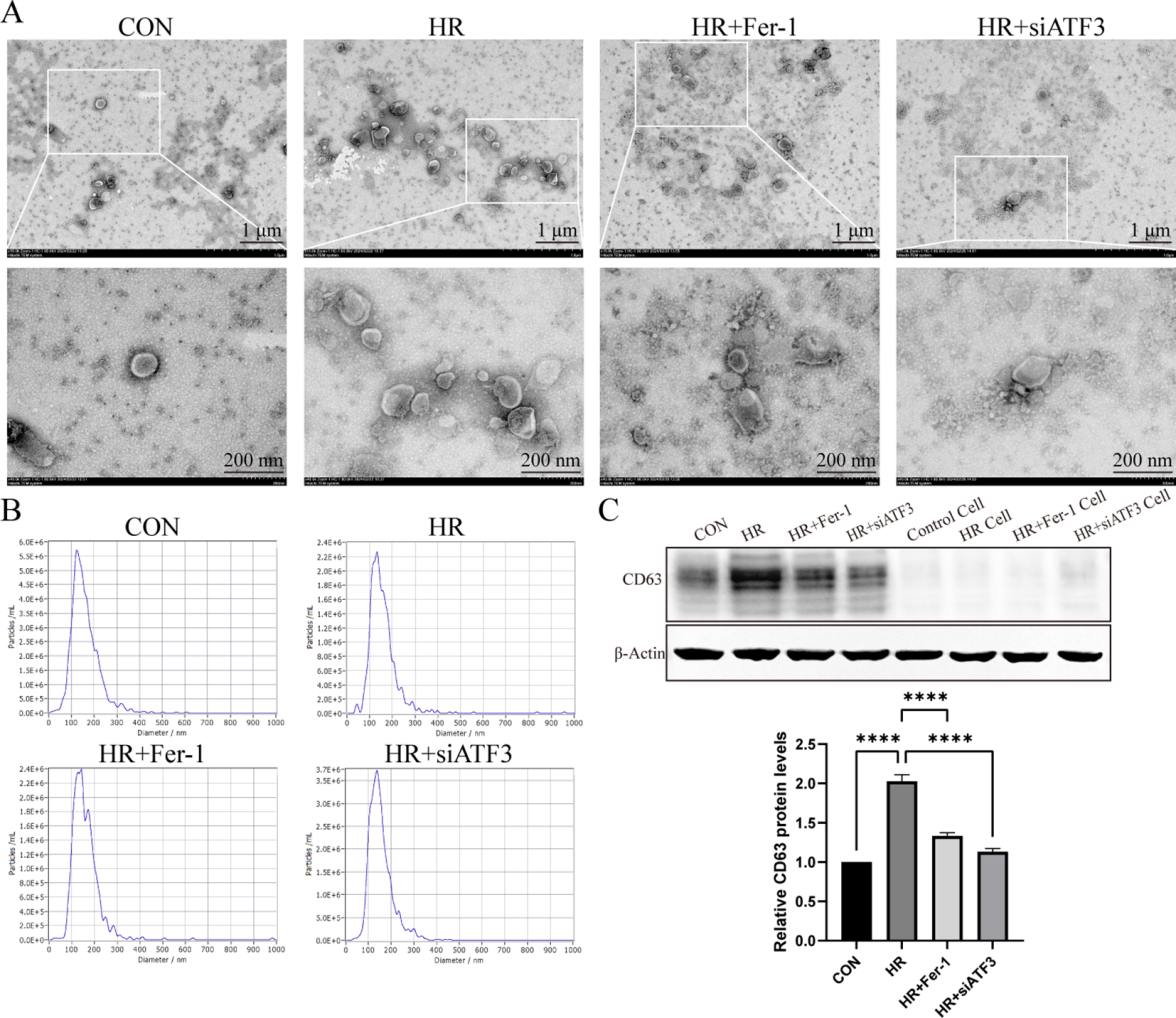
Characterization of exosomes derived from ferroptotic RTECs. **(A)** Representative pictures acquired by transmission electron microscopy (TEM). The scale bar represents 1 μm and 200 nm. **(B)** Average size distribution curve of exosomes as determined by nanoparticle tracking assay (NTA). **(C)** Relative level of protein expression of CD63 in the kidneys detected by Western blot. The protein level of sham group was normalized as 1. Data are expressed as means ± SD, ^****^P < 0.0001.

According to TEM (**Figure 5A**) and NTA (**Figure 5B**), the exosome particles of each group were about 50 nm to 200 nm in diameter, with elliptical spheres and central depression. Western blot analysis in **Figure 5C** revealed that these particles expressed CD63 expression, a widely recognized molecular marker of exosomes, was significantly increased in the HR group compared to the sham group, and Fer-1 treatment significantly reduced the exosomes content, as the same as HR+siATF3 groups. It should be mentioned that control refers to the exosomes isolated from the corresponding group, while control cell refers to the cell supernatant from the same group, which serves as a negative control to exclude potential contamination or infection from the supernatant. These results propose that HR can induce RTECs to generate more exosomes, and that these HR-RTECs are closely associated with ferroptosis, which can be hypothesized to be ferroptosis-related exosomes

### 3.6 Exosomes derived from ferroptotic RTECs promote M2 macrophage polarization

**Figure 6:**
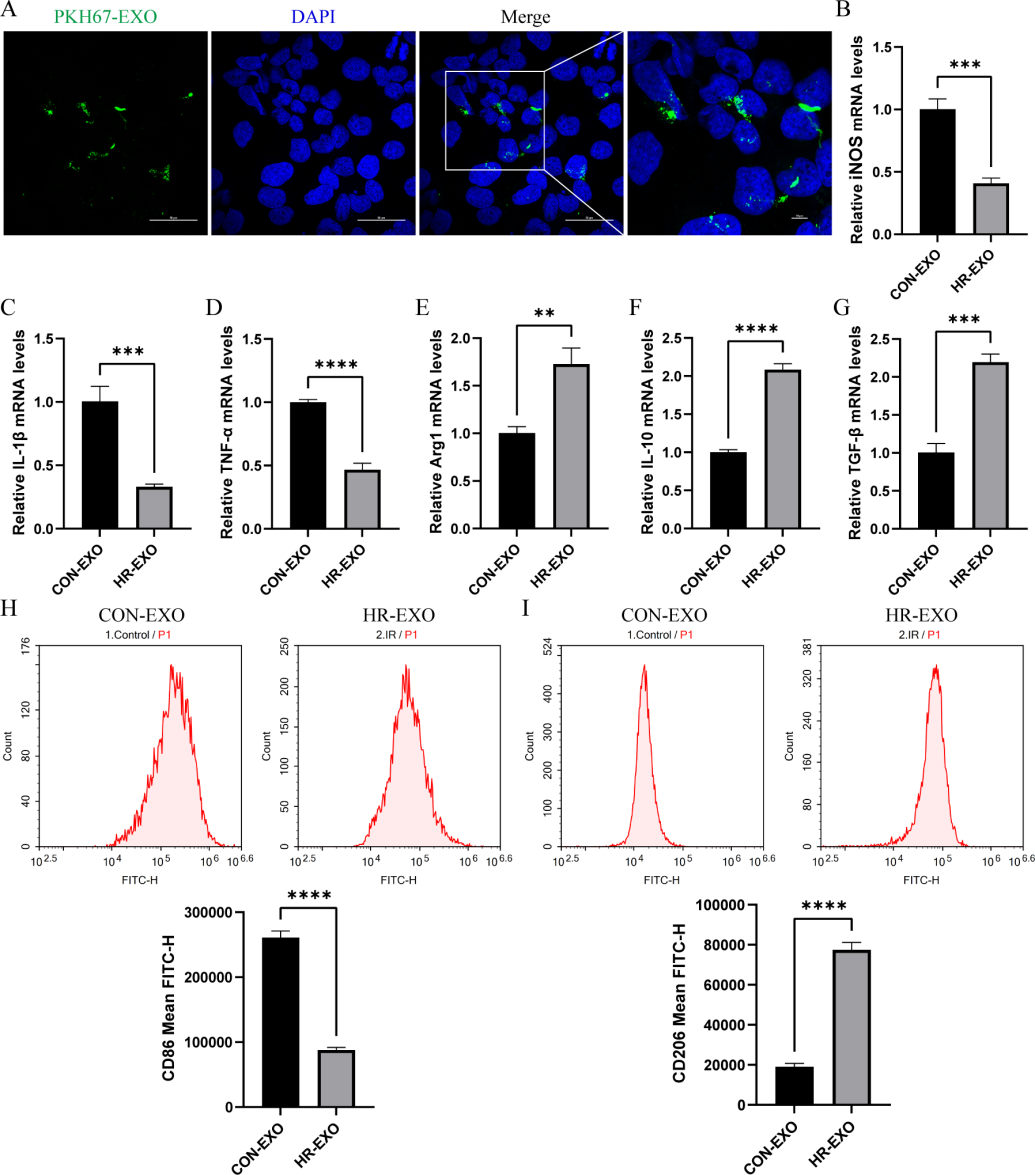
Exosomes derived from ferroptotic RTECs promote M2 polarization of macrophages. **(A)** Representative immunofluorescence staining images of RAW264.7 cells with exosomes derived from ferroptotic RTECs. The scale bar represents 50 μm and 10 μm. **(B-G)** Relative level of mRNA expression of TFRC and PTGS2 in RAW264.7 cells detected by RT-qPCR. **(H)** Mean FITC-H of CD86 antibody-labeled M1 phenotype macrophages in the CON-EXO and HR-EXO groups detected by flow cytometry. **(I)** Mean FITC-H of CD86 antibody-labeled M1 phenotype macrophages in the CON-EXO and HR-EXO groups detected by flow cytometry. Data are expressed as means ± SD, ^**^P < 0.01, ^***^P < 0.001, ^****^P < 0.0001.

Subsequently, we co-cultured exosomes derived from CON or HR groups with RAW264.7 cells (CON-EXO or HR-EXO). Intracellular transport of the exosomes into RAW264.7 cells was shown in **Figure 6A**, as PKH67-labeled exosomes (green fluorescence) were taken up by RAW264.7 cells and colocalized with DAPI-labeled nuclei (blue fluorescence). We detected the concentration of M1 markers (iNOS, IL-1β, and TNF-α) and M2 markers (Arg1, IL-10, and TGF-β) in the culture supernatant, and found that exosomes inhibited the production of iNOS, IL-1β, and TNF-α (**Figures 6B–D**), and enhanced Arg1, IL-10, and TGF-β (**Figures 6E–G**). Flow cytometry results also support that exosomes cause an increased proportion of CD206^+^ M2 phenotype macrophages (**Figures 6H and I**). In summary, these data indicated that exosomes secreted by ferroptotic RTECs promoted the polarization of macrophages towards M2. 

### 3.7 miRNA-seq analysis of exosomes derived from ferroptotic RTECs

**Figure 7:**
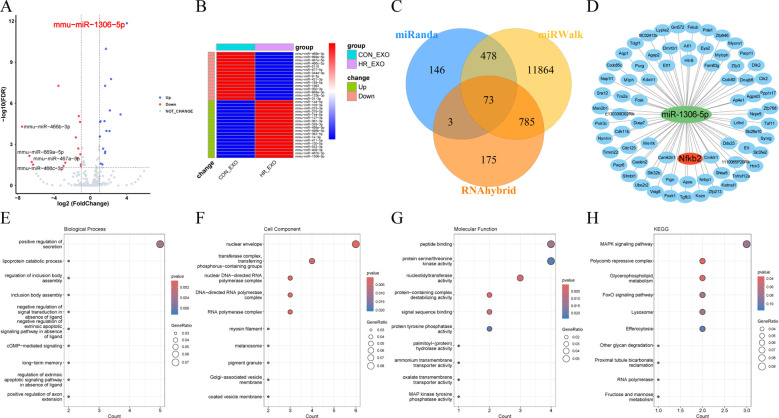
miRNA-seq analysis of exosomes derived from ferroptotic RTECs. **(A)** Volcano plots showing variable miRNAs in exosomes. **(B)** Heatmaps showing expression levels of differential miRNAs **(C)** Venn diagrams showing predicted target genes crossed over in miRWalk, miRanda and RNAhybrid. **(D)** Network diagram of miR-1306-5p and crossed predicted target genes shown by cytoscape software. **(E-H)** Dot plots showing the GO (biological process, cellular component and molecular function) and KEGG enrichment analysis results of miR-1306-5p target genes.

As shown in **Figure 7A**, the miRNA detection of exosomes secreted from HR-treated RTECs was analyzed. The heatmap showing the expression results of 33 miRNAs indicated that 18 miRNAs were significantly up-regulated in HR-EXO compared to CON-EXO, with the highest fold up-regulation of miR-1306-5p (*P* < 0.05; **Figure 7B**). As **Figures 7C, D** showed, potential targets of miR-1306-5p were predicted from three databases (miRWalk, miRanda, and RNAhybrid), and overlap analysis revealed 85 potential targets. As **Figures 7E–H** showed, they were significantly enriched in three major categories: BP (lipoprotein catabolic process, regulation of inclusion body assembly, cGMP-mediated signaling), CC (nuclear envelope, myosin filament, nuclear DNA-directed RNA polymerase complex), and MF (protein-containing complex destabilizing activity, nucleotidyltransferase activity, signal sequence binding). KEGG analysis revealed the major involvement in pathways such as Polycomb repressive complex, Glycerophospholipid metabolism, Other glycan degradation. 

## 4 Discussion

### 4.1 Interpretation of key findings

In this study, we report the following (1): ATF3/NRF2 pathway-mediated ferroptosis in RTECs acts as an important role in the IRI-associated RIF model, which is accompanied by M1/M2 ratio imbalance; (2) HR induces the secretion of ferroptosis-related exosomes by RTECs, and correlates with ATF3-mediated ferroptosis; (3) ferroptosis-related exosomes increase the relative proportion of M2 macrophages which secretes more fibrosis-relative factors. In conclusion, persistent activation of ATF3 by renal IR exacerbates ferroptosis in RTECs possibly by further inhibiting the Nrf2 pathway, and secretion of ferroptosis-related exosomes by RTECs contributes to hyperpolarization of M2 macrophages, which initiates IRI-related RIF occurrence.

### 4.2 Comparison with previous studies

Previous studies have shown that RTECs are the initiation site for the severe IRI-driven transition to RIF and that ferroptosis predominates in RTECs injury (23, 24). Renal tubules do not sensitize to necrotic apoptosis after selective removal of FAD or caspase-8, and the RIPK1 inhibitor necrostatin-1 (Nec-1) cannot protect freshly isolated tubules from hypoxic damage (25). An interesting feature of ferroptosis is that it can rapidly propagate between RTECs in a wave-like manner, which can be explained by the diffusion of the NADPH gradient formed by the reduced redox capacity within injured RTECs through intercellular junctions (26, 27). Notably, ferroptosis drives the accumulation of injured RTECs, which underlie renal interstitial fibers (14, 28). Therefore, this study focused on RTECs and demonstrated that ferroptosis occurs in RTECs to exacerbate RIF and that ferroptosis inhibition ameliorates renal tissue injury and attenuates renal fibrosis. Based on our previous analysis of ferroptosis-related genes before and after IR confirming that ATF3 is one of the genes abundantly expressed after IR (11), the present study also demonstrated that ATF3 is abundantly expressed in IRI-related RIF. ATF3 has been reported to inhibit system Xc- and deplete intracellular GSH, which promotes lipid peroxidation, contributing to ferroptosis (29). Furthermore, we demonstrated that ATF3 knockdown attenuated ferroptosis in RTECs, improved IRI-related RIF, and was accompanied by an increase in Nrf2 and HO-1 expressions. To summarize, one of our most important findings is the bioinformatic analysis-based confirmation that the ATF3/NRF2 signaling pathway regulates ferroptosis in RTECs, which not only refines a novel molecular mechanism of ferroptosis, but also provides a therapeutic target for the future treatment of renal fibrosis.

As is well known, ferroptosis not only contributes to the damage of renal parenchymal cells, but can also be recognized by immune cells, leading to a series of inflammation or specific reactions (30). During RIF, the most prominent immune cells are macrophages, which exhibit different polarization states at different stages due to phenotypic heterogeneity and functional diversity, playing an important role in the progression of IRI to CKD (31). iNOS^+^M1 macrophages are recruited into the kidney during early IRI, induced by exposure to IFNγ, LPS, TNF-α, or GM-CSF, and express pro-inflammatory cytokines such as IL-1β, TNF-α, and IL-6 (32). During the IRI recovery phase, IL-4 and/or IL-13 induce polarization of M2 macrophages, inhibite the expression of these pro-inflammatory markers, and activate the expression of arginase-1, mannose-receptor (MR), and IL-10. M2 macrophages produce pro-fibrotic factors (such as transforming growth factor (TGF) -β1, fibroblast growth factor 2 (FGF-2), platelet-derived growth factor (PDGF), and galactogenin 3) under the guidance of a variety of factors, which regulate epithelial and endothelial cell proliferation, myofibroblast activation, stem and tissue progenitor cell differentiation, and angiogenesis (33). Although M1 and M2 macrophages are the most commonly studied pro-inflammatory and anti-inflammatory phenotypes, macrophages are also shown to promote wound healing, pro-fibrosis, anti-fibrosis, pro-regression, and tissue regeneration (34, 35). Another important finding of the present study was that ferroptosis inhibition in RTECs involved the polarization of M2 macrophages, suggesting a potential relationship between ferroptotic RTECs and macrophages that needs to be further explored.

Exosomes are nanoscale (50 to 200 nm) extracellular vesicles, which, in addition to serving as biomarkers of disease, transmit a variety of signaling substances, including proteins, lipids, and sugars, as well as genetic signals such as DNA, mRNA, and microRNA (36). In acute and chronic renal injury models, RTECs release exosomes containing CCL2 mRNA and are taken up by macrophages, thereby exacerbating tubule interstitial inflammation (37). Hypoxic RTECs secret exosomes rich in miR-23a, which activate macrophages and promote tubulointerstitial inflammation (38). Exosomes from RTECs can activate fibroblasts by blocking the miR-21/PTEN/Akt pathway, thereby accelerating the development of renal fibrosis (39). miR-150-5p delivered by tubular cell-derived exosomes can negatively regulate the expression of cytokine signaling 1 inhibitor, which activates fibroblasts and prevents IRI from developing into renal fibrosis (20). Although the current studies began to explore exosomes secreted by RTECs affecting the progression of IRI-related RIF, few of them focused on the ferroptotic RTECs and exosomes. In the present study, we found that HR promoted cellular ferroptosis accompanied by increased exosome secretion, which could also be ameliorated by ferroptosis inhibitors, laterally revealing increased exosome release from ferroptotic RTECs. To further understand the functional specificity of ferroptosis-related exosomes in inducing M2 macrophage polarization, we identified exosome content components by miRNA-seq, with the highest expression being miR-1306-5p. Other previous studies have shown that miR-1306-5p was positively associated with adverse clinical outcomes in patients with acute heart failure (40, 41), and was also involved in other diseases such as glaucoma, human enamelogenesis imperfecta, and atherosclerosis (42–44). Of interest, miR-1306-5p has a target gene, Nfkb2, which is one of the important components of the transcription factor NF-kB that promotes the release of various cytokines and chemokines (e.g., TNFα, IL1β, IL6, IL12, CXCL9, etc.) from M1 macrophages via the non-classical NF-κB pathway (45). Therefore, we hypothesized that miR-1306-5p binding to Nfkb2 via base complementary pairing inhibits Nfkb2 expression at the post-transcriptional level, ultimately leading to an increase in the relative proportion of M2 macrophages. Anyway, our study demonstrated a novel mechanism involved in IRI-related RIF from the perspective of iron metabolism, i.e., exsomes derived from ferroptotic RTECs contained miR-1306-5p and were indeed picked up by macrophages, which were transformed into fewer M1 macrophages and more M2 macrophages that secreted more pro-fibrotic factors. However, this study has not explored the specific mechanism by which miR-1306-5p causes the decrease in the relative ratio of M1/M2 macrophages after miRNA-seq analysis, which will be further investigated by our next study.

### 4.3 Study limitations and future directions

Our study has several limitations. First, the lack of clinical data is a major limitation. Although our experiments were conducted using murine and cell-based models, these findings have yet to be validated in human clinical samples. The absence of clinical data limits the generalizability and translational potential of our results, and future research incorporating clinical samples is needed to confirm their relevance to human disease. Second, the exact mechanism through which miR-1306-5p regulates macrophage polarization remains unclear. While our study suggests that miR-1306-5p is involved in this process, further investigation is required to identify its direct targets and the specific pathways through which it exerts its effects on macrophage polarization. This will be an important direction for future research.

### 4.4 Conclusion

Briefly, this study has confirmed that ATF3 promotes ferroptosis possibly by regulating Nrf2, and that ferroptotic cells-derived exosomes carrying miR-1306-5p led to an increase in the relative proportion of M2 macrophages, which ultimately exacerbates RIF following renal IRI. Therefore, ATF3 is a potential therapeutic target for the treatment of renal IRI-related RIF.

## Data Availability

The raw data supporting the conclusions of this article will be made available by the authors, without undue reservation.
