# Correction: Exosomes secreted by ATF3/Nrf2-mediated ferroptotic renal tubular epithelial cells promote M1/M2 ratio imbalance inducing renal interstitial fibrosis following ischemia and reperfusion injury

**DOI:** 10.3389/fimmu.2025.1642215

**Published:** 2025-07-15

**Authors:** Qiao Tang, Jiatao Xie, Yifei Wang, Chong Dong, Qian Sun

**Affiliations:** ^1^ Department of Anesthesiology, Renmin Hospital of Wuhan University, Wuhan, China; ^2^ The First Clinical College of Wuhan University, Wuhan, China; ^3^ Organ Transplantation Center, Tianjin First Central Hospital, Tianjin, China; ^4^ Tianjin Key Laboratory for Organ Transplantation, Tianjin, China

**Keywords:** ischemia and reperfusion, renal interstitial fibrosis, ferroptosis, activation transcription factor 3, exosome, macrophages

There was a mistake in article text as published. ATF3 was erroneously mentioned as AKF3 at various instances throughout the article, although all experimental data, analyses, and conclusions in the study correctly refer to ATF3. All instances of “AKF3” have been replaced with “ATF3”.

The original version of this article has been updated.

